# Promoter Methylation Status of Ras-Association Domain Family Members in Pheochromocytoma

**DOI:** 10.3389/fendo.2015.00021

**Published:** 2015-02-19

**Authors:** Antje M. Richter, Tobias Zimmermann, Tanja Haag, Sara K. Walesch, Reinhard H. Dammann

**Affiliations:** ^1^Institute for Genetics, University of Giessen, Giessen, Germany

**Keywords:** pheochromocytoma, tumor suppressor, DNA methylation, epigenetics, RASSF

## Abstract

Pheochromocytomas (PCCs) are rare neuroendocrine tumors that arise from the medulla of the adrenal gland or the sympathetic ganglia and are characterized by the secretion of catecholamines. In 30–40% of patients, PCCs are genetically determined by susceptibility genes as various as RET, VHL, and NF1. We have analyzed the Ras-association domain family members (RASSFs) in PCCs regarding their inactivating promoter hypermethylation status. Previously, we reported a promoter methylation in PCC for the first family member RASSF1A. Promoter hypermethylation of CpG islands leads to the silencing of the according transcript and is a common mechanism for inactivation of tumor suppressors. In this study, we observed inactivating DNA modifications for the RASSF members RASSF2, RASSF5A, RASSF9, and RASSF10, but not for the members RASSF3, RASSF4, RASSF5C, RASSF6, RASSF7, and RASSF8. The degree of promoter methylation was 19% for RASSF2, 67% for RASSF5A, 18% for RASSF9, and 74% for RASSF10. Interestingly, the degree of hypermethylation for RASSF10 in hereditary PCCs was 89 vs. 60% in sporadic PCCs. A similar but less dramatic effect was observed in RASSF5A and RASSF9. Including all RASSF members, we found that of 25 PCCs, 92% show promoter methylation in at least in one RASSF member. In 75% of the hereditary PCC samples, we found two or more methylated RASSF promoters, whereas in sporadic PCCs only 46% were observed. In summary, we could show that in PCC several RASSF members are strongly hypermethylated in their promoter regions and methylation of more than one RASSF member occurs in the majority of PCCs. This adds the inactivation of genes of the RASSF tumor suppressor family to the already known deregulated genes of PCC.

## Introduction

Pheochromocytomas (PCCs) are rare catecholamine-secreting tumors derived from chromaffin cells and 10% of PCCs are malignant. Tumors are malign when the capsule is invaded or metastases occur. The hormone imbalance affects the cardiovascular system leading to hypertension and other metabolic processes. PCC treatment involves treatment of hypertension and surgical removal of the tumor. (1, 2) The occurrence peaks between the ages of 20–40 and up to 30–40% occur due to germline mutations of 11 known susceptibility genes like HIF2A (hypoxia-inducible factor 2α) or SDH (succinate dehydrogenase) (3). The syndromes familial multiple endocrine neoplasia (MEN) type 2A/2B, von Hippel-Lindau disease, and neurofibromatosis are also associated with PCC development (4). Regarding RASSF1A, which is frequently inactivated in different tumor entities (5, 6), we have reported its inactivation in the tumor entity of PCC (7). Tumor suppressors act antiproliferatively and are known to be negatively regulated by their CpG island promoter hypermethylation, leading to the inactivation of the according transcript in neoplasia (8). Our focus lies on the tumor suppressors of the Ras-association domain family (RASSF) containing 10 members (RASSF1A to RASSF10), which are frequently inactivated via the methylation of their CpG island promoter (9). The RASSFs were first described in 2000, by the characterization of the first family member RASSF1A (10). The family members are characterized by the presence of the Ras-association domain (C-terminus or N-terminus) and additional protein–protein interaction domains [SARAH (11) or coiled-coil domains]. Their functions range from microtubule network interaction, RAS interaction, Hippo pathway involvement, cell cycle regulation to apoptosis induction (9, 12). Interestingly, mice studies exist for several RASSFs and suggest an involvement in tissue or organ size control (13–17). Due to our earlier studies of RASSF1A inactivation in PCC (7) and the known contribution of tumor suppressor inactivation to cancer development (8), we were interested in the possibility that inactivation of more than one RASSF member occurs in PCC. Their inactivation could contribute to tumor formation of PCC and we therefore used the combined bisulfite restriction analysis (COBRA) methylation analysis to study the promoter methylation status of the RASSF members RASSF2, RASSF3, RASSF4, RASSF5A, RASSF5C, RASSF6, RASSF7, RASSF8, RASSF9, and RASSF10.

## Experimental

### CpG island prediction, PCR product size, and digestion products

Promoter regions of RASSF2, RASSF3, RASSF4, RASSF5A, RASSF5C, RASSF6, RASSF7, RASSF8, RASSF9, and RASSF10 were analyzed for their CpG islands by CpG plot *http://www.ebi.ac.uk/Tools/seqstats/emboss_cpgplot/* and ucsc genome browser https://genome.ucsc.edu/. Except for RASSF6 and RASSF9, all members show prominent CpG islands. Primers for COBRA methylation analysis were designed to frame at least one restriction site for *Taq*I enzyme on bisulfite treated and therefore fully converted DNA (unmethylated cytosine to uracil). The resulting PCR products lengths, digestion products, and PCR conditions are listed in Table S1 in Supplementary Material.

### Pheochromocytoma and controls

Primary tissues were previously published (7). All patients signed informed consent at initial clinical investigation. The study was approved by a local ethic committee (Martin-Luther University, Halle, Germany).

### Methylation analysis by COBRA

DNA was isolated by phenol-chloroform extraction. Two micrograms of genomic DNA from PCC was bisulfite treated (12 μl of 0.1 M hydroquinone, 208 μl of 1.9 M sodium metabisulfite, and pH 5.5 with NaOH) and incubated over night at 50°C. Then DNA was purified using MSB Spin PCRapace (STRATEC Molecular, Berlin, Germany), eluted in 50 μl H_2_O and followed by 10 min incubation with 5 μl of 3 M NaOH at 37°C. DNA was then precipitated with 100% ethanol and 7.5 M ammonium acetate and resolved in 1× TE buffer. Two hundred nanograms were subsequently used for 25 μl PCR reaction with COBRA primers. The PCR product was either mock digested or digested with 0.5 μl of *Taq*I (Fermentas GmbH, St.Leon-Rot, Germany) 1 h at 65°C and resolved on 2% TBE gel together with mock control (18, 19). Digestion of the PCR product indicates an underlying CpG methylation at the analyzed restriction site. We used an *in vitro* methylated (*ivm*) DNA as a positive control for COBRA. For *in vitro* methylation of genomic DNA, we used M.SssI methylase (NEB, Frankfurt, Germany) (20). Human fibroblasts (HF) were used as a normal unmethylated control cell line.

## Results

**Figure 1:**
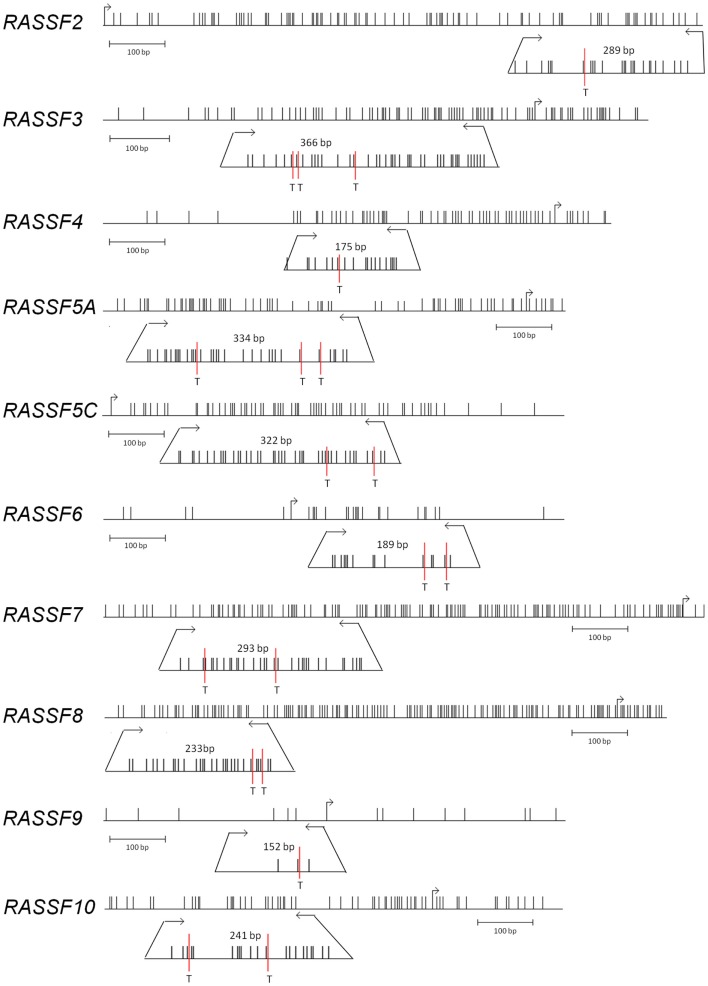
**Genomic RASSF promoter regions**. RASSF family members RASSF2, RASSF3, RASSF4, RASSF5A, RASSF5C, RASSF6, RASSF7, RASSF8, RASSF9, and RASSF10 are shown with their promoter region. Single CpGs are represented as vertical black lines, restriction enzyme *Taq*I recognition sites are marked with T, and bent arrows indicate transcriptional start sites. Horizontal arrows mark the COBRA PCR products obtained after bisulfite treatment of DNA and according PCR fragment size. Hundred base pair standard is shown below each promoter region. Graphics were generated with the python.vs.cobra program (https://launchpad.net/python.vs.cobra).

In the current study, we have analyzed a total of 25 PCC patient samples of which 13 were sporadic and 12 hereditary (Table 1). We have studied the promoter methylation status of RASSF2, RASSF3, RASSF4, RASSF5A, RASSF5C, RASSF6, RASSF7, RASSF8, RASSF9, and RASSF10. DNA promoter methylation is an indicator of epigenetic inactivation of tumor suppressor genes (TSGs) (8). The CpG island sizes are 1.1 kb for RASSF2, 1.1 kb for RASSF3, 0.5 kb for RASSF4, 1.2 kb for RASSF5A, 0.5 kb for RASSF5C, 0.2 kb for RASSF6, 1 kb for RASSF7, 1.2 kb for RASSF8, not predicted for RASSF9, and 2.3 kb for RASSF10. We used the COBRA method for promoter methylation analysis, which is based on bisulfite treatment of genomic DNA for conversion of unmethylated cytosines to uracils. The following PCR amplifies the defined region and is followed by digestion with *Taq*I restriction enzyme (Figure 1). PCR fragments still containing restriction sites (methylated and therefore unchanged CpG) are digested and fragments no longer containing restriction sites (earlier unmethylated CpG) remain undigested. Therefore, a digestion of the PCR product indicates a methylation of the according genomic CpGs that were analyzed. Localization of COBRA primers within the analyzed CpG promoter region, transcription start site, CpG enrichment, restriction site in PCR product, and PCR product size are shown for each analyzed family member (Figure 1). Apparently, RASSF6 and RASSF9 show little CpG island presence, but were analyzed as well.

**Table 1:** **RASSF promoter methylation in sporadic (SP) and hereditary (MP) pheochromocytoma**.

	Nr	Age	Sex	Status	R1A	R2	R3	R4	R5A	R5C	R6	R7	R8	R9	R10
1	SP17	49	f	b	1	n.a.	0	0	0	0	0	n.a.	n.a.	n.a.	n.a.
2	SP2	36	f	b	1	0	0	0	1	0	0	0	0	0	n.a.
3	SP1	72	m	b	1	1	0	0	1	0	0	0	0	0	1
4	SP25	54	m	m	1	1	0	0	1	0	0	0	0	0	0
5	SP4	49	m	b	1	n.a.	n.a.	n.a.	n.a.	0	n.a.	0	0	0	n.a.
6	SP11	52	f	m	0	0	0	0	0	0	0	0	0	0	0
7	SP6	34	m	m	0	0	0	0	1	0	0	0	0	0	0
8	SP13	44	m	b	0	n.a.	n.a.	n.a.	1	0	n.a.	0	0	1	1
9	SP15	20	f	b	0	0	n.a.	n.a.	1	0	n.a.	0	0	1	0
10	SP5	42	f	b	0	0	0	0	0	0	0	0	0	0	1
11	SP22	44	m	b	0	0	0	0	0	0	0	0	0	0	1
12	SP26	64	f	b	0	n.a.	0	0	n.a.	n.a.	0	0	0	0	1
13	SP14	75	f	b	0	0	0	0	1	0	0	0	0	0	1
14	MP19	38	f	b	1	1	n.a.	0	0	0	0	0	0	1	1
15	MP24	31	m	b	1	0	n.a.	n.a.	1	0	n.a.	0	0	1	1
16	MP18	22	f	b	1	n.a.	0	0	0	0	0	0	0	0	1
17	MP8	45	m	b	1	n.a.	0	0	1	0	0	0	0	0	1
18	MP23	31	f	b	1	0	0	0	0	0	0	0	0	0	n.a.
19	MP21	29	f	b	1	0	0	0	1	0	0	0	0	0	n.a.
20	MP10	47	f	b	1	0	0	0	1	0	0	0	0	0	0
21	MP16	36	m	b	0	0	0	0	1	0	0	0	0	0	1
22	MP3	23	m	b	0	n.a.	n.a.	n.a.	n.a	n.a.	n.a.	0	0	0	n.a.
23	MP9	20	f	b	0	0	0	0	1	0	0	0	0	0	1
24	MP12	18	f	b	0	n.a.	n.a.	n.a.	n.a.	n.a.	n.a.	n.a.	n.a.	n.a.	1
25	MP20	51	m	b	0	n.a.	n.a.	0	1	0	0	n.a.	n.a.	n.a.	1

*f, female; m, male; b, benign; m, malign; 1(gray), methylated promoter; 0, unmethylated promoter; n.a., not analyzed*.

**Figure 2:**
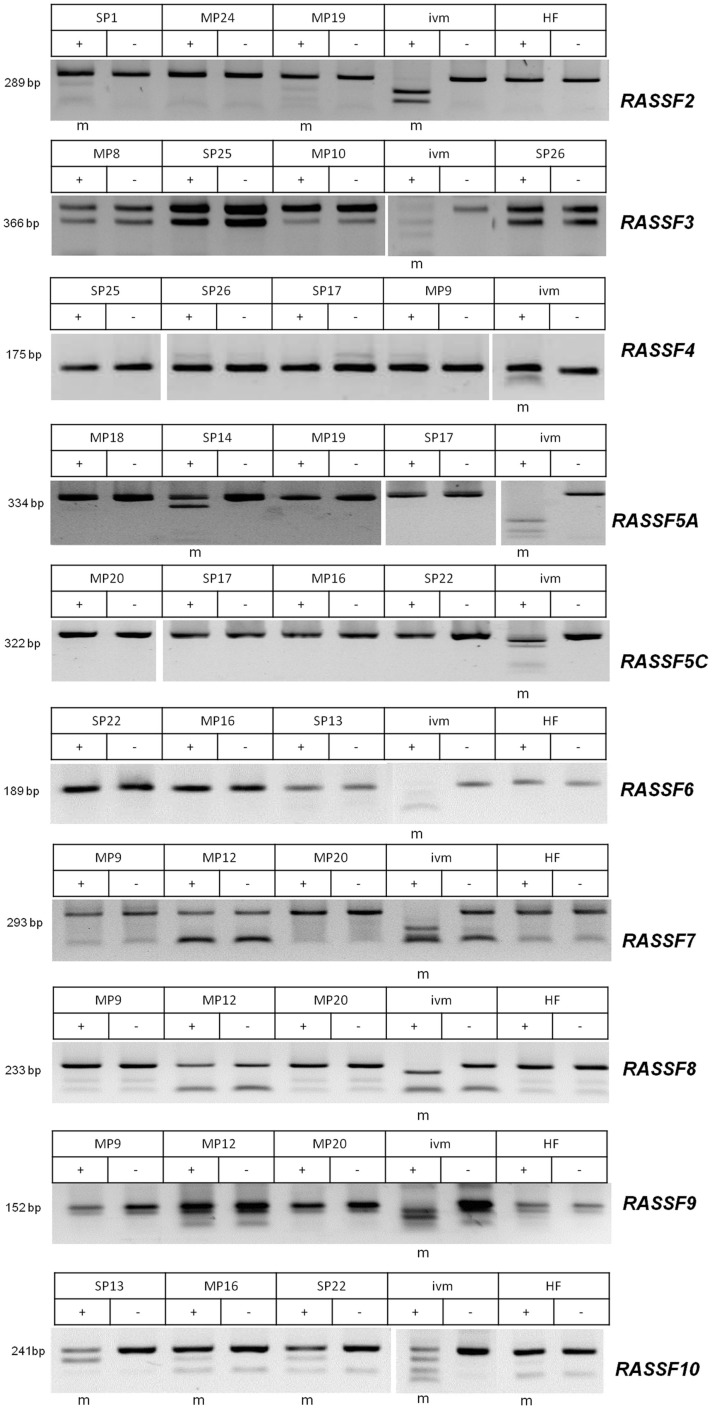
**Methylation analyses by COBRA for RASSF2, −3, −4, −5A, −5C, −6, −7, −8, −9, and −10**. Methylation analysis by COBRA method for RASSF2, −3, −4, −5A, −5C, −6, −7, −8, −9, and −10 was performed and PCR product sizes are shown. PCR products were digested with *Taq*I enzyme (+) or mock digested (−). Sporadic (SP) and hereditary (MP) PCCs are exemplarily shown for each RASSF together with *in vitro* methylated (*ivm*) positive control sample and human fibroblasts (HF) as normal cell control. Methylated samples are marked (m) below each gel.

Our results show promoter methylation for RASSF2, RASSF5A, RASSF9, and RASSF10, but not for any of the other analyzed members of the family (Figure 2). RASSF2 shows in 3 of 16 samples (19%), a methylation, RASSF5A in 14 of 21 samples (67%), RASSF9 in 4 out of 22 samples (18%), and RASSF10 in 14 out of 19 samples (74%). For RASSF3 0/17, RASSF4 0/19, RASSF5C 0/22, RASSF6 0/19, RASSF7 0/22, and RASSF8 0/22 and therefore no samples showed methylation of their promoter region (Tables 1 and 2). For COBRA analysis, we used *ivm* DNA as a positive control for methylation detection after bisulfite treatment. In this study, HF serve as a negative control for promoter methylation analysis. This non-cancerous cell line reaches senescence after few passages like normal tissues (21).

**Table 2:** **Summary of RASSF promoter methylation**.

	R1A^a^	R2	R3	R4	R5A	R5C	R6	R7	R8	R9	R10
Samples methylated/total	12/25 (48%)	3/16 (19%)	0/17 (0%)	0/19 (0%)	14/21 (67%)	0/22 (0%)	0/19 (0%)	0/22 (0%)	0/22 (0%)	4/22 (18%)	14/19 (74%)
Sporadic samples methylated/total	5/13 (38%)	2/9 (22%)	0/10 (0%)	0/10 (0%)	7/11 (64%)	0/12 (0%)	0/10 (0%)	0/12 (0%)	0/12 (0%)	2/12 (17%)	6/10 (60%)
Hereditary samples methylated/total	7/12 (58%)	1/7 (14%)	0/7 (0%)	0/9 (0%)	7/10 (70%)	0/10 (0%)	0/9 (0%)	0/10 (0%)	0/10 (0%)	2/10 (20%)	8/9 (89%)

*^a^Dammann et al. (2005)*.

**Figure 3:**
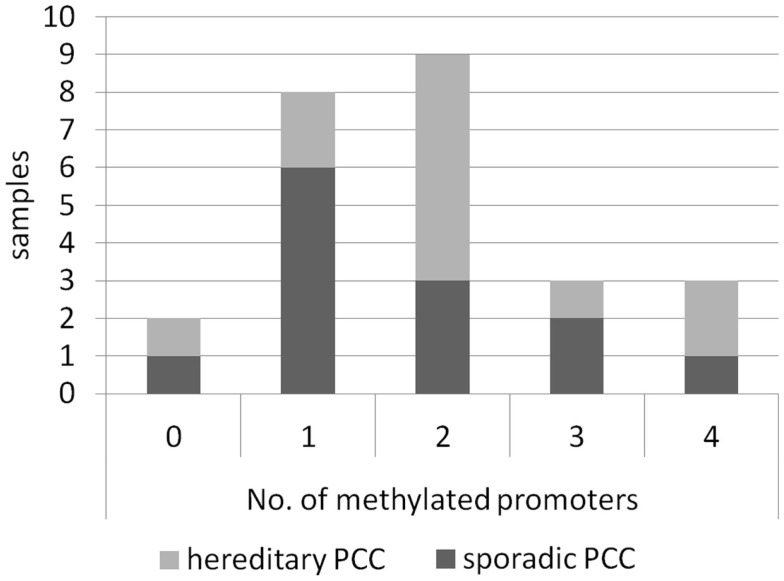
**Occurrence of co-methylated RASSF promoters in PCCs**. Samples of sporadic (*n* = 13) and hereditary (*n* = 12) PCCs are shown regarding their number of methylated RASSF promoters for all RASSFs. For details, see Table 1.

We have analyzed sporadic as well as hereditary PCC samples and found promoter hypermethylation in both subsets of PCC for RASSF2, RASSF5A, RASSF9, and RASSF10 (Table 1). Interestingly, the promoter of RASSF10 showed an increase in methylation from 60% of sporadic samples to 89% of hereditary PCCs. A similar but less dramatic effect was observed in RASSF5A and RASSF9. Furthermore, correlation analysis neither showed an association of methylation status of RASSF2, RASSF5A, RASSF9, and RASSF10 with age, sex, bilateral localization, noradrenalin/adrenalin expression nor the presence of p16, VHL, MSH2, MLH1 promoter methylation, which we studied earlier (7). We compared the number of co-methylated promoters from all RASSFs in the 25 PCCs, which included RASSF1A (7). We found that only two samples remained unmethylated for all RASSF promoters (Figure 3). The other 23 PCC samples showed one up to four methylated RASSF promoters per sample. In all PCCs, we observed a distribution of eight samples with one promoter methylation, nine samples with two methylated promoters, and six samples with three or four methylated promoters (Figure 3). Six sporadic PCC samples showed one promoter methylation and six samples had two or more promoters being methylated. In hereditary PCCs, we found however only two samples with one promoter methylation vs. nine samples with two or more methylated promoters (Figure 3).

## Discussion

The aim of our study was the comparative analysis of RASSFs regarding their promoter methylation status in PCC. The tumor suppressor family RASSF consists of 10 members, with the first member RASSF1 being the best studied one. Tumor suppressors are known to be inactivated by CpG island promoter hypermethylation (8) and we and others have already shown the RASSF1A promoter methylation in PCC (7, 22). A later study even correlated RASSF1A promoter methylation with PCC malignancy (23). In this study, we analyzed the family members RASSF2, RASSF3, RASSF4, RASSF5A, RASSF5C, RASSF6, RASSF7, RASSF8, RASSF9, and RASSF10, of which some have been characterized as tumor suppressors (9, 12). So far, no other study analyzed PCC and the promoter methylation of our studied RASSFs except for one publication showing no promoter methylation for RASSF5A in primary PCCs (23). However, a significant downregulation of RASSF5A has been reported in PCC (19), although the relevant mechanism was not identified. We have studied sporadic and hereditary PCCs regarding their promoter methylation status of RASSFs. We observed a strong hypermethylation in PCCs for RASSF2 (19%), RASSF5A (67%), RASSF9 (18%), and RASSF10 (74%), but not in the other family members (Table 2). Interestingly, the degree of methylation was higher in hereditary PCCs when compared to sporadic PCCs for RASSF5A (70 vs. 64%) and RASSF10 (89 vs. 60%; Table 1), which is in accordance with the RASSF1A (58 vs. 38%) methylation status (7).

Regarding all analyzed PCCs, we found that 92% show promoter methylation of at least one RASSF member. Additionally, we observed the co-methylation of several RASSF promoters in the same PCC sample. The comparison of sporadic to hereditary PCCs shows an increase in the number of samples with two or more methylated RASSF promoters from 46% of sporadic to 75% of hereditary PCCs (Figure 3). In a hereditary PCC background, an additional RASSF inactivation seems a driver of tumor formation and underlines the importance of expression regulation of RASSFs. It would be interesting to analyze this positive association in a larger cohort of PCCs. We found an inverse correlation of RASSF10 methylation with malignancy and infiltration of the capsule, which very likely must be attributed to the rather small sample number. Interestingly, we observed a promoter methylation for RASSF9 despite the absence of a predictable CpG island. It can be presumed that even the RASSF9 promoter methylation plays a role in its inactivation.

The family members that showed no methylation in our PCC analysis were studied earlier in different cancer types with differing results. Regarding RASSF3 and RASSF4, very little data exist, but it was reported that RASSF3 is epigenetically inactivated in somatotroph adenomas (24) and RASSF4 in human tumor cells (25). RASSF5C is the 3′ transcript of the RASSF5 locus (26), similar to the isoforms RASSF1A and C, containing a CpG island, but hardly ever being methylated and thereby inactivated (18, 26–28). Due to the small size of the RASSF6 CpG island, an epigenetic regulation is rather unlikely. However, in neuroblastoma, RASSF6 and RASSF7 showed a promoter methylation and RASSF6 promoter methylation was correlated with an unfavorable outcome (29). RASSF8 methylation was reported in a small subset of leukemia (30). In further studies, we would like to address a larger set of PCCs regarding promoter methylation of RASSFs and if possible include normal adrenal gland tissue as well as the PCC cell line PC12. It will be fascinating to further study the functional contribution of inactivated RASSFs for PCC development or progression. It will be especially interesting to dissect why several RASSF members are inactivated in the same samples and therefore presumably missing. This co-inactivation in PCC suggests distinct and separate pathways through which RASSF1A, RASSF2, RASSF5A, RASSF9, and RASSF10 function in the normal adrenal gland. It seems plausible that inactivation of several members of the RASSF family contributes to tumor formation and progression. It could also underline that RASSFs are not all redundant and interchangeable. Knockdown experiments of each of the members will be of valuable use and studies using the available knockout mice could indicate the contribution of co-inactivation of the RASSFs.

## Conclusion

In our study, we are the first to show the promoter methylation status of all RASSFs in sporadic and hereditary PCC. We have found promoter hypermethylation for the family members RASSF1A, RASSF2, RASSF5A, RASSF9, and RASSF10, but not for RASSF3, RASSF4, RASSF5C, RASSF6, RASSF7, or RASSF8. Our observations show co-appearance of CpG promoter hypermethylation of several RASSF members in PCC, especially in the hereditary PCCs.

## Conflict of Interest Statement

The authors declare that the research was conducted in the absence of any commercial or financial relationships that could be construed as a potential conflict of interest.

## Supplementary Material

The Supplementary Material for this article can be found online at http://www.frontiersin.org/Journal/10.3389/fendo.2015.00021/abstract

## Abbreviations

COBRA, combined bisulfite restriction analysis; PCC, pheochromocytoma; RASSF, Ras-association domain family; TSG, tumor suppressor gene.
