# Supplementary material for: Promoter Methylation Status of Ras-Association Domain Family Members in Pheochromocytoma
Source: Front Endocrinol (Lausanne). 2015 Feb 19;6:21. doi: 10.3389/fendo.2015.00021 (PMC4333862; doi:10.3389/fendo.2015.00021)
Supplement: Supplementary file 1 [file Table1.docx]

Supplementary Table 1: COBRA Primers

| **gene** | **PCR** | **upper primer** | **lower primer** | **PCR product (bp)** | **digestion products (bp) *Taq*1** |
| --- | --- | --- | --- | --- | --- |
| RASSF2 | 1.PCR | 5’GATGGGAAGGYGTTTTTATTTTATTTT | 5’CCCAACCACCTCAAACACCAACTCC | 289 | 111+178 |
| RASSF3 | 1.PCR | 5‘GGGTAATTTTTATTTATTTTTATAGGATTTTG | 5‘ACCCCRCCTCCCTAAACCCC | 366 | 92+7+75+192 |
| RASSF4 | 1.PCR | 5‘AAATGTTTTGGGTTTTATTGATAAGTTTTTTT | 5‘ACCAAATATCCCCAAACCATTATTCAACTC | 264 | - |
|  | 2.PCR | 5‘GTAGCGGTTTTTGTTGGAAGTTTAGGAGTT | 5‘ACCAAATATCCCCAAACCATTATTCAACTC | 175 | 70+105 |
| RASSF5A | 1.PCR | 5‘GGATAGTTTTGTTAGTTTTTGGAGGTATT | 5‘ACCCTAAACCTTCAACCCTACCTCTT | 363 | - |
|  | 2.PCR | 5‘GGATAGTTTTGTTAGTTTTTGGAGGTATT | 5‘CTTACCAATCACTTTCCCCAACAC | 334 | 94+140+26+74 |
| RASSF5C | 1.PCR | 5‘GGTTTTGAGGAATTTTGTAGAGGAA | 5‘AAAAAAAATAAACACCCCTCCCC | 380 | - |
|  | 2.PCR | 5‘AGGAAGTGGTTTTAGAATTGTTTTA | 5‘TAAACCCCTAACTCTAAACCCC | 322 | 221+63+38 |
| RASSF6 | 1.PCR | 5‘TTAGTTGAGTTATGTTTTGGGAGGAGAT | 5‘TTATTCACACTATAAACAAAAAAACCCTTT | 189 | 69+75+45 |
| RASSF7 | 1.PCR | 5‘GGGATTTTAAGTTAGAGATTTTTTTTGGG | 5‘CCCCTAACACCCCTATTAACTACTCAAC | 331 | - |
|  | 2.PCR | 5‘GTTATATTAGAAGGTTGTTGAGGAAGT | 5‘CCCCTAACACCCCTATTAACTACTCAAC | 293 | 57+93+143 |
| RASSF8 | 1.PCR | 5‘TTTAAATTTAGTTTTTGGAGGGGGGATGG | 5‘CCAACcCTACATTATAAAAACTTTTAAAAC | 322 | - |
|  | 2.PCR | 5‘GTAGAGGGTAGTTTGATAAGTTTTAGTTT | 5‘CCAACcCTACATTATAAAAACTTTTAAAAC | 233 | 178+12+43 |
| RASSF9 | 1.PCR | 5‘AGGAGTTGGGGGATATTTATTTTTAATTT | 5‘AAAAAACTACCAACCCCACAAACATAA | 152 | 96+56 |
| RASSF10 | 1.PCR | 5’ATAAGTAGAGGAGTTAGTAGGTTAAAGGAGA | 5‘AAATACAAAAAACTCAAAACCCAAACCC | 241 | 49+91+101 |
